# Tuning IL-2 signaling by ADP-ribosylation of CD25

**DOI:** 10.1038/srep08959

**Published:** 2015-03-10

**Authors:** Sophie Teege, Alexander Hann, Maria Miksiewicz, Cary MacMillan, Björn Rissiek, Friedrich Buck, Stephan Menzel, Marion Nissen, Peter Bannas, Friedrich Haag, Olivier Boyer, Michel Seman, Sahil Adriouch, Friedrich Koch-Nolte

**Affiliations:** 1 Institute of Immunology, University Medical Center, 20246 Hamburg, Germany; 2 Department of Clinical Chemistry, University Medical Center, 20246 Hamburg, Germany; 3 Department of Radiology, University Medical Center, 20246 Hamburg, Germany; 4 Normandy University, Institute for Research and Innovation in Biomedicine, 76183 Rouen, France; 5 Inserm, U905, 76000 Rouen, France; 6 Rouen University Hospital, Department of Immunology, 76000 Rouen, France

## Abstract

Control of immunologic tolerance and homeostasis rely on Foxp3^+^CD4^+^CD25^+^ regulatory T cells (Tregs) that constitutively express the high affinity receptor for Interleukin-2, CD25. Tregs proliferate in response to injections of IL-2/anti-IL-2 antibody complexes or low doses of IL-2. However, little is known about endogenous mechanisms that regulate the sensitivity of CD25 to signaling by IL-2. Here we demonstrate that CD25 is ADP-ribosylated at Arg35 in the IL-2 binding site by ecto-ADP-ribosyltransferase ARTC2.2, a toxin-related GPI-anchored ecto-enzyme. ADP-ribosylation inhibits binding of IL-2 by CD25, IL-2- induced phosphorylation of STAT5, and IL-2-dependent cell proliferation. Our study elucidates an as-yet-unrecognized mechanism to tune IL-2 signaling. This newly found mechanism might thwart Tregs at sites of inflammation and thereby permit a more potent response of activated effector T cells.

Interleukin 2 (IL-2) activates multiple immune cells, but mounting evidence indicates that its primary function is to support the generation and survival of Tregs that inhibit immune responses and prevent autoimmune diseases^1^,^2^,^3^,^4^. IL-2 signals through high and low affinity cell surface receptors^5^,^6^,^7^. CD25, the α-chain of the IL-2 receptor, is constitutively associated with lipid rafts^8^. Assembly of the heterotrimeric high affinity receptor complex is initiated by binding of IL-2 to CD25, followed by recruitment of CD122 and CD132. In the absence of CD25, the β and γ chains form a receptor with a 10–100 fold lower affinity for IL-2^9^. This low affinity receptor is expressed by naïve T cells, memory CD8^+^ T cells, and NK cells. Both receptors signal via phosphorylation of STAT5 (signal transducer and activator of transcription 5)^10^,^11^,^12^. The crystal structure of IL-2 in complex with its receptor revealed the residues of CD25 involved in binding of IL-2^6^,^7^, including a prominent arginine doublet (R35R36) (Supplementary Fig. 1a, b).

T cell subsets are differentially modulated by IL-2 signaling, depending on the concentration of IL-2 and on the expression of the IL-2 receptor subunits. CD25 is constitutively expressed at high levels by Tregs and IL-2 signaling through CD25 is crucial for the generation, survival and function of these cells^1^,^2^,^3^,^13^,^14^,^15^. By efficient consumption of IL-2, Tregs can deprive neighboring T cells of this cytokine^16^,^17^. Recent studies show that *in vivo* treatment with low doses of IL-2 or with IL-2 in complex with anti-IL-2 antibodies can suppress immune-mediated diseases by inducing the expansion of Tregs^17^,^18^,^19^,^20^,^21^,^22^. IL-2-specific antibodies can prolong the serum half-life of IL-2 by inhibiting renal filtration of the low molecular weight cytokine^23^. Intriguingly, different IL-2/anti-IL-2 antibody complexes induce distinct responses *in vivo*, depending on the epitope of IL-2 bound by the particular antibody: mouse IL-2 in complex with mAb (monoclonal antibody) JES6-1A12 or human IL-2 in complex with mAb 5344 induce the preferential expansion of Tregs, whereas mouse IL-2 in complex with mAb S4B6 or human IL-2 in complex with mAb-602 induce the preferential expansion of CD8^+^ cytotoxic T cells and NK cells^23^,^24^,^25^,^26^,^27^,^28^. These findings raise the question whether endogenous mechanisms exist that could similarly modulate IL-2 signaling *in vivo*.

NAD^+^-dependent ADP-ribosylation is one of the posttranslational modifications regulating protein functions^29^,^30^. NAD^+^ (nicotinamide adenine dinucleotide) is released from cells during inflammation and acts as a signaling molecule that alerts immune cells to sites of tissue damage^31^,^32^,^33^,^34^. The NAD^+^-dependent toxin-related ADP-ribosyltransferase ARTC2.2 is a GPI-anchored enzyme expressed on the cell membrane of naïve T cells and Foxp3^+^CD4^+^CD25^+^ regulatory T cells (Tregs)^35^,^36^,^37^. ARTC2.2 transfers the ADP-ribose moiety from NAD^+^ to arginine residues of other raft-associated membrane proteins^35^,^38^. For example, ADP-ribosylation of the P2X7 ion channel at R125 induces gating of the ion channel^38^. ADP-ribosylation of cell surface proteins can be monitored by autoradiography or by antibody-based immunoassays (Supplementary Fig. 1c, d).

Here we uncover ADP-ribosylation of CD25 as a novel mechanism that controls IL-2 signaling following exposure of Tregs to extracellular NAD^+^. Using affinity purification of ADP-ribosylated cell surface proteins and mass spectrometry, we identify CD25 as a major target of ARTC2.2. We show that ARTC2.2-catalyzed, NAD^+^-dependent ADP-ribosylation of CD25 inhibits IL-2 binding, IL-2-induced phosphorylation of STAT5, and IL-2-dependent cell-proliferation. We propose that ADP-ribosylation of CD25 provides an endogenous mechanism to tune IL-2 signaling in response to NAD^+^ released from injured cells during inflammation and tissue damage. By inhibiting formation of the high affinity IL-2 receptor naturally expressed by regulatory T cells, this mechanism could allow the preferential expansion of activated effector T cells in inflammatory settings.

## Results

### Identification of CD25 as a major target of ARTC2.2

**Figure 1:**
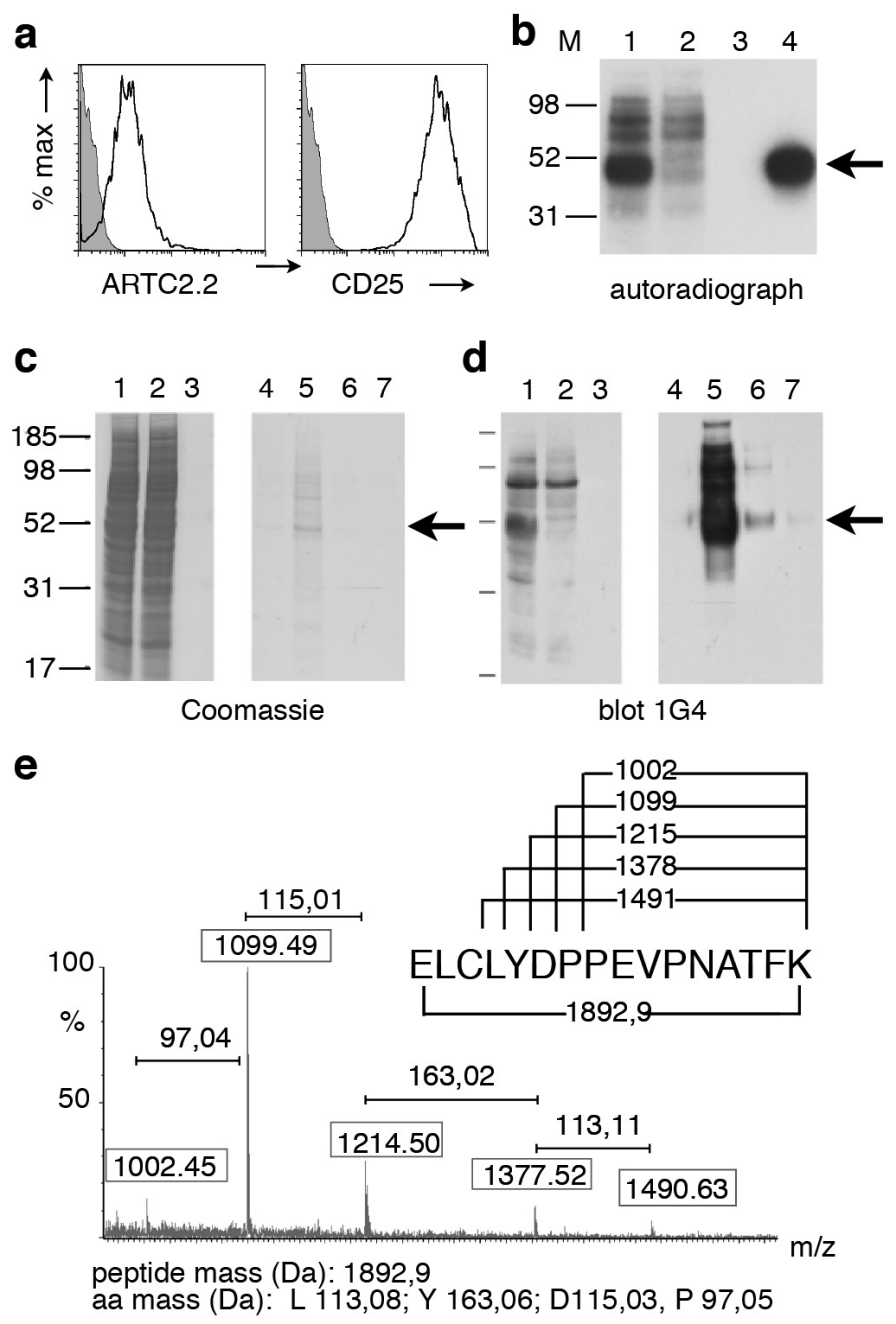
Immunoprecipitation and mass spectrometry analyses identify CD25 as a major ADP-ribosylation target on YAC-1 cells. (a) YAC-1 cells were stained with fluorochrome-conjugated mAbs specific for ARTC2.2 and CD25 and analyzed by flow cytometry. (b) YAC-1 cells were incubated with ^32^P-NAD^+^. Cell lysates were subjected to immunoprecipitation, proteins were size fractionated by SDS-PAGE, and incorporated radioactivity was detected by autoradiography. Lanes 1, 2: cell lysates before and after precipitation of CD25; lane 3: control precipitation with protein G, lane 4: precipitation with anti-CD25 (PC61). (c–e) YAC-1 cells were incubated with etheno-NAD^+^. Etheno-ADP-ribosylated proteins were purified from the cell lysate using an affinity column with etheno-adenosine specific mAb 1G4. Bound proteins were eluted in four fractions with etheno-adenosine. Proteins in the cell lysate (lane 1), column flow through (lane 2), wash (lane 3), and eluates (lanes 4–7) were size fractionated by SDS-PAGE. Total protein was visualized by Coomassie staining (c), etheno-ADP-ribosylated proteins were visualized in a parallel immunoblot analysis with mAb 1G4 (d). The 50 kD band in lane 5 (panel c) was excised from the gel and subjected to trypsin digestion and nanospray mass spectrometry. MALDI-TOF spectrum of a fragmented tryptic peptide of 1892,9 kD (e). Boxed numbers indicate the masses of individual peptide peaks, numbers above brackets indicate the mass differences between adjacent peptide peaks. The amino acid sequence of the N-terminal peptide of CD25 is shown on top with numbers corresponding to the masses of the respective peptide fragments. Results are representative of three independent experiments.

The YAC-1 lymphoma cell line constitutively expresses ARTC2.2 and CD25 (Fig. 1a). Incubation of these cells with ^32^P-NAD^+^ resulted in the labeling of several bands, including a prominent band of 50 kD (Fig. 1b, lane 1, arrowhead). Similar results were obtained when etheno-NAD^+^ was used as substrate (Fig. 1d, lane 1, arrowhead). Affinity purification of etheno-ADP-ribosylated proteins from YAC-1 cell lysates (Fig. 1c, d) followed by mass spectrometry analyses (Fig. 1e) identified the protein in this band as CD25. Immunoprecipitation analyses with CD25-specific antibodies verified the identity of the protein in this prominent 50 kD band as CD25, i.e. this band was depleted from cell lysates and was recovered in the immunoprecipitate (Fig. 1b, compare lanes 1, 2, and 4).

### ARTC2.2 ADP-ribosylates CD25 at R35 in the IL-2 binding site

**Figure 2:**
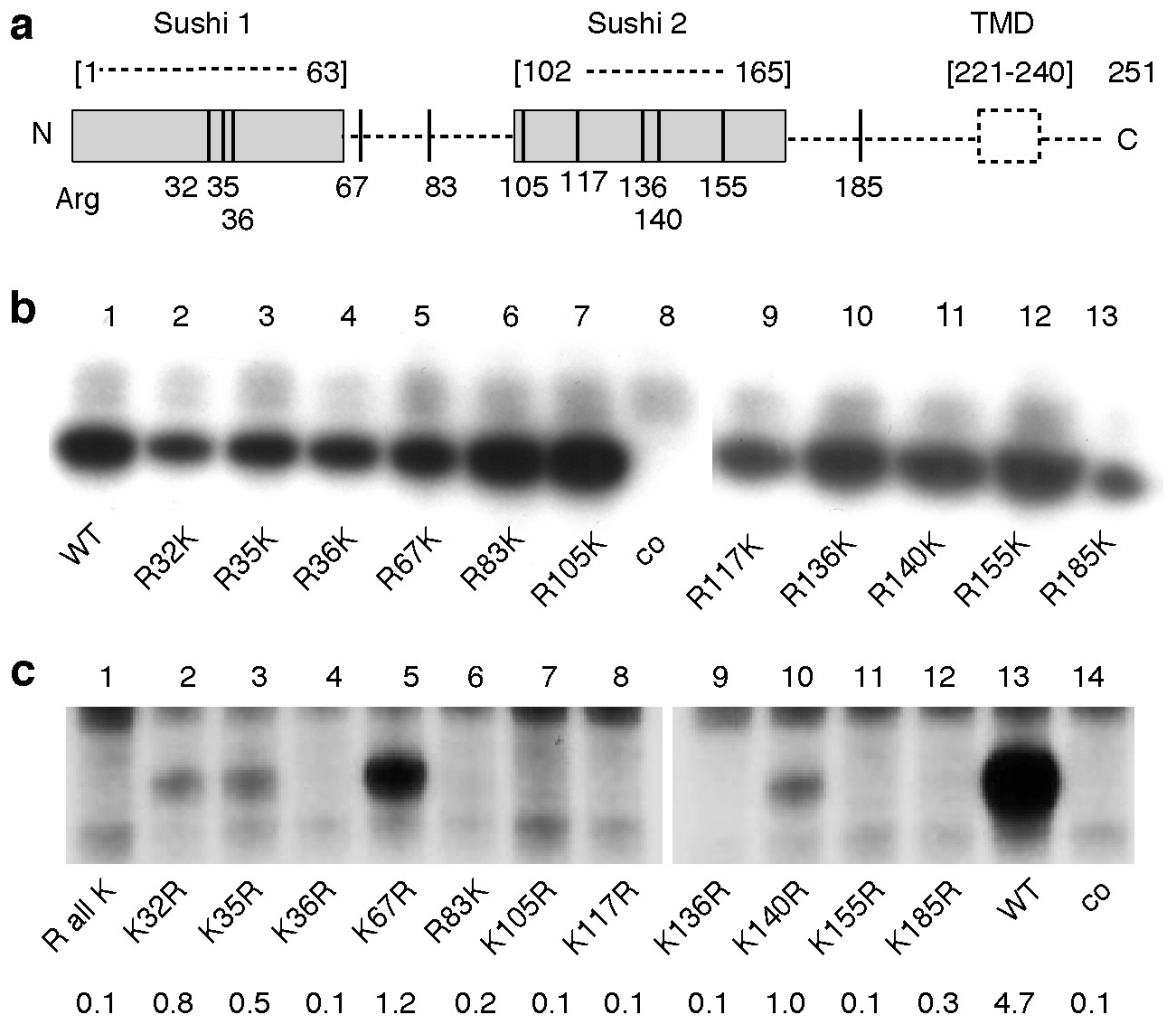
Identification of of R32, R35, R67, and R140 as the ADP-ribosylation sites on CD25. (a) Schematic diagram of the 11 arginine residues in the extracellular domains of human CD25. Numbers correspond to the position within the amino acid sequence of the native protein, i.e. after removal of the signal peptide. Dashed lines indicate residues not visible in the 3D-structure of the IL-2 receptor complexed with IL-2 (2erj). TMD: transmembrane domain. (b, c) HEK cells were co-transfected with expression vectors for ARTC2.2 and either a non-ADP-ribosylated control antigen (co), wild type CD25 (WT), or CD25 variants. Analyzed mutants of CD25 include single R > K mutants (*B*), a synthetic construct in which all arginine residues in the extracellular domain were replaced by lysine (RallK), and variants of RallK carrying individual K > R back mutations (c). 48 hours post transfection, cells were incubated with ^32^P-NAD^+^. Radiolabeled proteins were detected by SDS-PAGE autoradiography. Results are representative of four independent experiments. Numbers indicate the relative labeling intensity of individual bands (mean from four repeats).

The 3D-structure of human IL-2 in complex with its heterotrimeric receptor reveals that all eight arginine residues in the visible portion of the extracellular domain of CD25 are localized on the surface of the protein and thus are potentially accessible for ADP-ribosylation (Supplementary Fig. 1b, Fig. 2a). In order to test which of these arginines could be ADP-ribosylated by ARTC2.2, we individually substituted each of the arginine residues with lysine by site-directed mutagenesis. In addition, we purchased a synthetic cDNA construct of CD25, in which all 11 arginines were substituted by lysine (RallK). In this construct, we individually substituted each lysine with arginine, thereby generating variants of CD25 carrying only a single arginine residue. Each mutant was co-transfected into HEK (human embryonic kidney) cells together with ARTC2.2. 40 h post transfection, cell surface expression levels were assessed by flow cytometry using ARTC2.2 and CD25-specific mAbs (Supplementary Fig. 2). Parallel aliquots of cells were incubated with ^32^P-labeled NAD^+^. Radiolabeling of CD25 and other cell surface proteins was assessed by SDS-PAGE autoradiography (Fig. 2b, c).

The results revealed that each of the single R > K mutants of CD25 still served as a target for ARTC2.2, suggesting that CD25 is ADP-ribosylated at more than one site (Fig. 2b). Similar results were obtained for mouse CD25 (Supplementary Fig. 3). This is reminiscent of previous reports on the ADP-ribosylation of P2X7 and of defensin 1, both of which are ADP-ribosylated at more than one arginine^38^,^39^. RallK, the mutant in which all arginines were replaced by lysine, did not incorporate any radiolabel (Fig. 2c, lane 1) despite robust expression on the cell surface (Supplementary Fig. 2). This finding confirms that ADP-ribosylation of CD25 by ARTC2.2 is arginine-specific. Four of the RallK reversion mutants carrying a single arginine residue were radiolabeled by ARTC2.2, indicating that these sites can, in principle, serve as ADP-ribosylation sites for ARTC2.2 (Fig. 2c, R32, R35, R67, R140). Three of these residues (R32, R35, R140) are conserved in mouse CD25 (Supplementary Fig. 4). The fourth residue (R67) is located in a linker between the two sushi domains that is not visible in the 3D structure. The stronger labeling of the R67 revertant indicates that this residue is more accessible for ARTC2.2 than the other revertants. R35 is part of an arginine doublet (R35/R36), as are the primary ADP-ribosylation sites on P2X7 and defensin 1^38^,^39^. R35 is located within the IL-2 binding interface (Supplementary Fig. 1), R32 and R140 are located on the edges of the first and second Sushi domains, respectively, and R67 is located on the flexible linker between the two sushi domains that is not visible in the 3D-structure of the IL-2 receptor complex (Supplementary Fig. 4).

### ADP-ribosylation inhibits binding of mAb 7D4 but not mAb PC61

**Figure 3:**
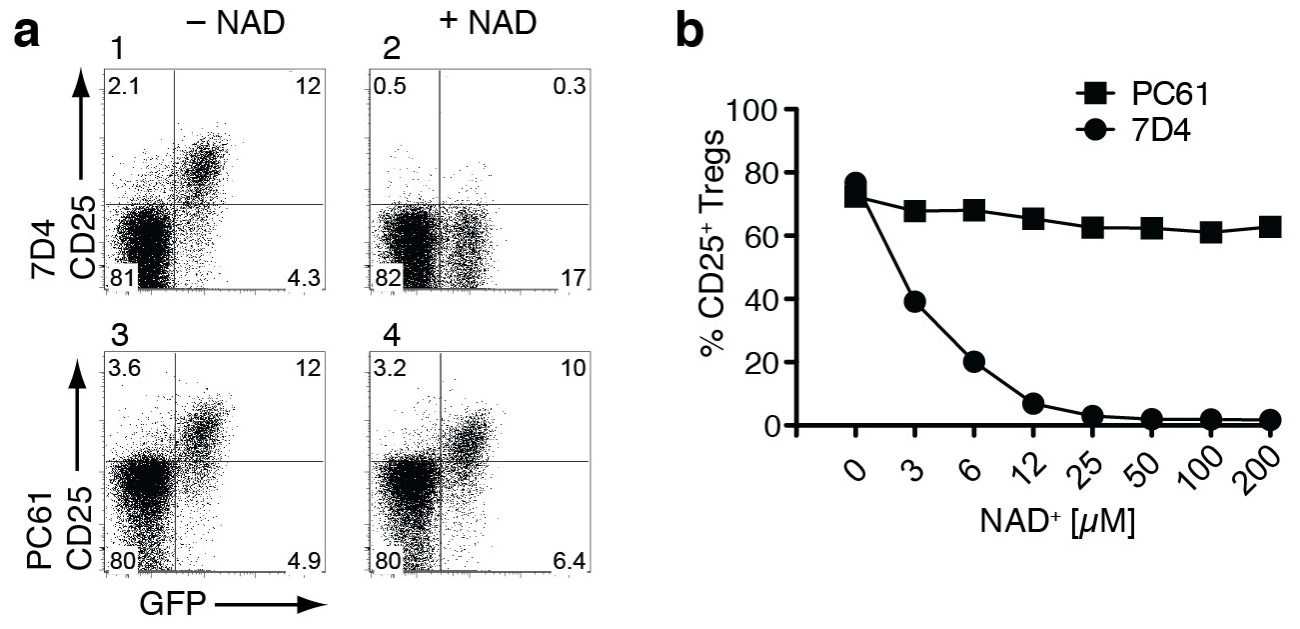
ADP-ribosylation inhibits binding of anti-CD25 mAb 7D4 but not of mAb PC61. Splenocytes from DEREG mice were incubated in the absence or presence of the indicated concentrations of NAD^+^ before staining with fluorochrome-conjugated mAbs directed against CD4 and CD25 (mAb PC61 or mAb 7D4). Gating was performed on CD4^+^ cells. (a) Representative dot plots of cells incubated in the absence or presence of 12 μM NAD^+^. (b) Percentages of CD4^+^GFP^+^ cells staining with anti-CD25 mAbs plotted as a function of the concentration of added NAD^+^. Results are representative of two independent experiments.

ADP-ribosylation results in the attachment of a bulky residue and imparts a local charge change (from a positively charged arginine to a negatively charged ADP-ribosylarginine)^40^. It is, therefore, conceivable that ADP-ribosylation of CD25 at or near an antibody binding-site could hinder antibody binding. In order to address this question, we incubated mouse splenocytes in the absence or presence of NAD^+^ followed by staining with the CD25-specific mAbs PC61 and 7D4 (Fig. 3). The results reveal a NAD^+^ dose-dependent inhibition of staining by 7D4 but little if any effect of NAD^+^ on the binding of PC61 (Fig. 3b), indicating that ADP-ribosylation affects the epitope recognized by 7D4 but not the epitope recognized by PC61.

### ADP-ribosylation inhibits IL-2 binding and IL-2-dependent proliferation of CTLL-2 cells

**Figure 4:**
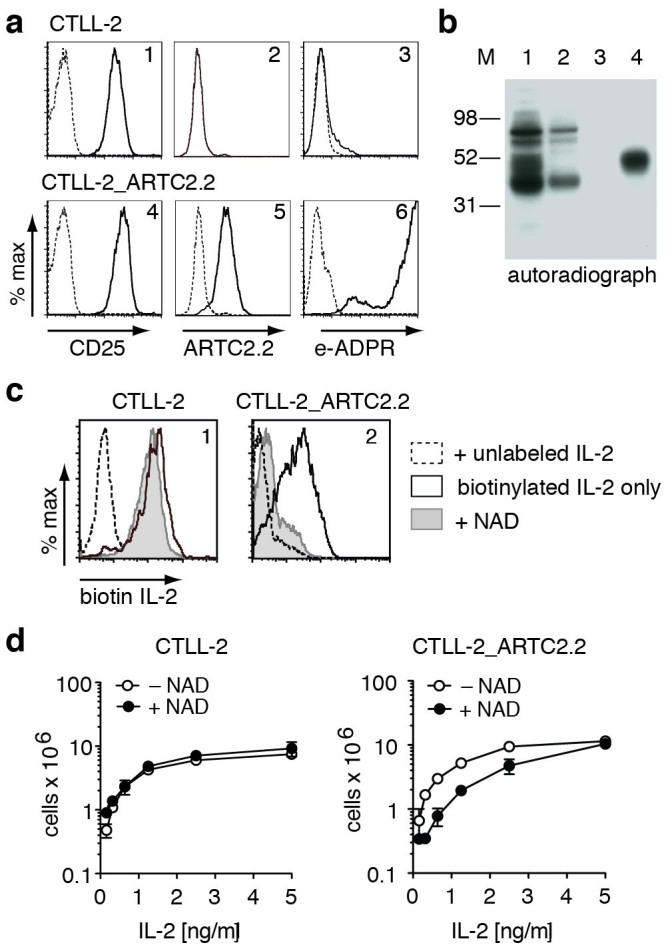
ADP-ribosylation inhibits binding of IL-2 and IL-2-dependent proliferation of ARTC2.2-transfected CTLL-2 lymphoma cells. (a) Untransfected parental and ARTC2.2-transfected CTLL-2 cells were incubated with etheno-NAD^+^ and stained with fluorochrome-conjugated mAbs specific for CD25, ARTC2.2, and etheno-adenosine and analyzed by flow cytometry. (b) CTLL-2^ARTC2.2^ cells were incubated with ^32^P-NAD^+^ and cell lysates were subjected to immunoprecipitation with anti-CD25 and SDS-PAGE autoradiography as in Fig. 1b. Lanes 1, 2: lysates before and after precipitation of CD25, lane 3: control precipitation with protein G, lane 4: precipitation with anti-CD25. (c) CTLL-2 and CTLL-2^ARTC2.2^ cells were pre-incubated in the absence or presence of NAD^+^ or unlabeled IL-2. Cells were then incubated with biotinylated IL-2 before addition of fluorochrome-conjugated streptavidin and analysis by flow cytometry. (d) CTLL-2 and CTLL-2^ARTC2.2^ cells were seeded in a 24 well culture plate (3 × 10^4^/well) and incubated for four days in medium containing the indicated concentrations of IL-2. Medium lacking or containing NAD^+^ was added every 12 hours. Cell numbers were assessed by flow cytometry with the aid of Trucount beads (BD). Results are representative of two independent experiments.

In order to determine whether ADP-ribosylation affects binding of IL-2 and IL-2-dependent cell proliferation, we turned to the IL-2-dependent CTLL-2 cell line (Fig. 4). In contrast to YAC-1 cells, CTLL-2 cells do not proliferate in the absence of exogenous IL-2. Moreover, also in contrast to YAC-1 cells, CTLL-2 cells do not express ARTC2.2 and show little if any cell surface ART activity (Fig. 4a, panels 2 and 3). Following stable transfection with ARTC2.2, CTLL-2^ARTC2.2^ cells acquire the capacity to ADP-ribosylate cell surface proteins (Fig. 4a, panel 6), with CD25 as a prominent target (Fig. 4b). The availability of ART-negative and ARTC2.2-expressing CTLL-2 cells permits direct assessment of the effects of ADP-ribosylation on cellular functions, e.g. by comparing the responses of CTLL-2^ARTC2.2^ and parental CTLL-2 cells to extracellular NAD^+^.

In order to assess whether ADP-ribosylation of CD25 affects binding of IL-2, CTLL-2 and CTLL-2^ARTC2.2^ cells were incubated for 15 min with biotinylated IL-2 in the absence or presence of unlabeled IL-2 or NAD^+^. Binding of biotinylated IL-2 was then assessed with APC-conjugated streptavidin (Fig. 4c). The results show that pre-incubation with NAD^+^ inhibits binding of IL-2 by CTLL-2^ARTC2.2^ cells but not by parental CTLL-2 cells (grey shaded histograms). In contrast, control analyses performed by pre-incubating cells with unlabeled IL-2 blocked binding of biotinylated IL-2 by both, CTLL-2^ARTC2.2^ and parental CTLL-2 cells (Fig. 4c, dashed histograms).

In order to assess whether ADP-ribosylation affects IL-2-dependent cell proliferation, CTLL-2 and CTLL-2^ARTC2.2^ cells were depleted of IL-2 by washing with PBS followed by incubation for 6 hours in IL-2 free medium. Cells were then seeded in 24-well plates in medium containing serial dilutions of IL-2 and cells were cultivated further in the absence or presence of NAD^+^ for 3.5 days. Cell numbers were assessed by flow cytometry using Trucount beads (Fig. 4d). The results show that NAD^+^ does not affect the proliferation of parental CTLL-2 cells whereas it markedly inhibits the proliferation of CTLL-2^ARTC2.2^ cells at low and intermediate doses of IL-2 (below 5 ng/ml). These results suggests that ADP-ribosylation of cell surface proteins interferes with IL-2 binding and with IL-2 signaling.

### ADP-ribosylation inhibits IL-2-induced phosphorylation of STAT5

**Figure 5:**
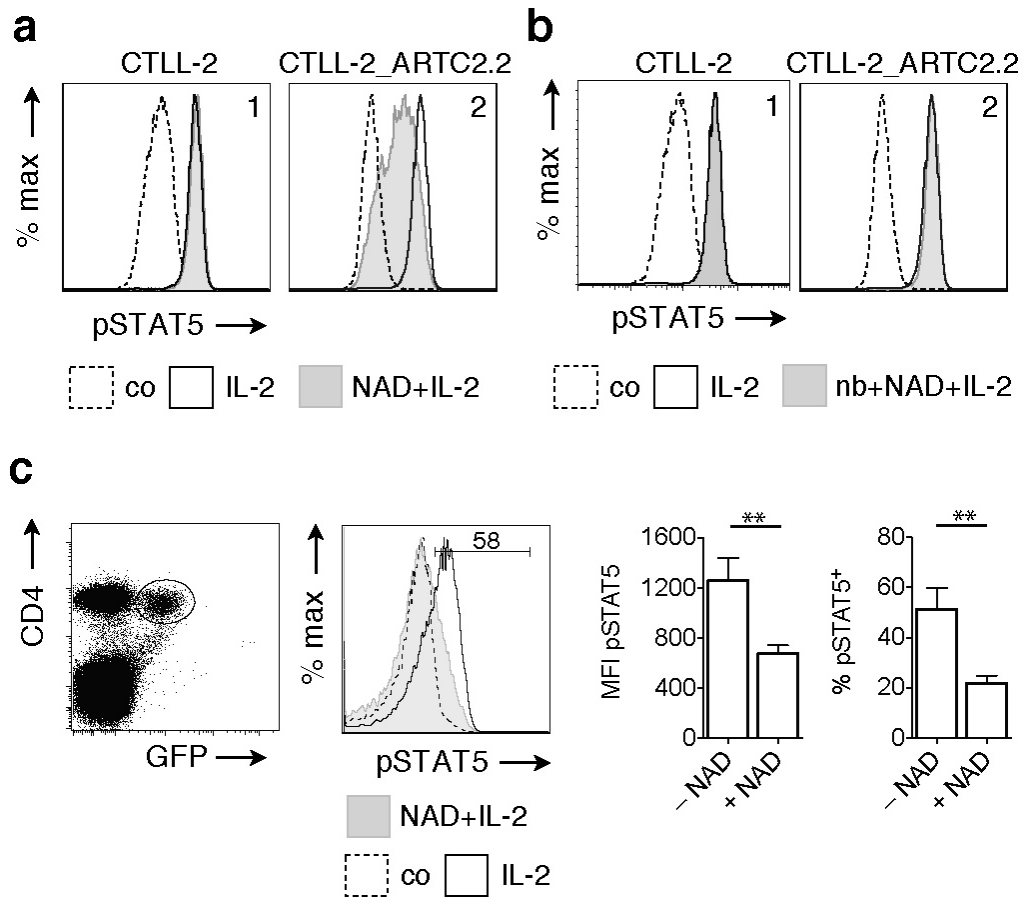
ADP-ribosylation inhibits IL-2-induced phosphorylation of STAT5 by CTLL-2^ARTC2.2^ cells and by ARTC2^+/+^ Tregs. (a) CTLL-2 and CTLL-2^ARTC2.2^ cells were pre-incubated in the absence or presence of NAD^+^ before stimulation with IL-2 for 15 min. Cells were fixed and stained with fluorochrome-conjugated antibodies against phosphorylated STAT5 (pSTAT5). Control cells were incubated without NAD^+^ or IL-2. (b) CTLL-2 and CTLL-2^ARTC2.2^ cells were pre-incubated with the ARTC2.2-blocking nanobody s+16a (nb) before addition of NAD^+^ and stimulation with IL-2. (c) Tregs were FACS-sorted from wildtype DEREG mice and incubated in the absence or presence of NAD^+^ before stimulation with IL-2 and staining for pSTAT5. Results are representative of two independent experiments.

Binding of IL-2 is known to initiate a signal transduction pathway resulting in activation of STAT5 by phosphorylation. The latter can be visualized by flow cytometry with mAb 47 specific for pSTAT5 (Fig. 5). In order to assess whether ADP-ribosylation interferes with IL-2-induced phosphorylation of STAT5, CTLL-2 and CTLL-2^ARTC2.2^ cells were treated with NAD^+^ and then analyzed by FACS for IL-2-induced phosphorylation of STAT5 (Fig. 5a). The results show that treatment with extracellular NAD^+^ inhibits IL-2-induced phosphorylation of STAT5 in case of CTLL-2^ARTC2.2^ cells (Fig. 5a, panel 2, shaded histogram) but not of parental CTLL-2 cells (Fig. 5a, panel 1). Preventing ARTC2.2-catalyzed ADP-ribosylation of cell surface proteins with the ARTC2.2-blocking nanobody s+16a^41^ completely prevented inhibition of STAT5 phosphorylation in CTLL-2^ARTC2.2^ cells in the presence of NAD^+^ (Fig. 5b, panel 2), confirming that the effect of NAD^+^ on STAT5 phosphorylation is dependent on ARTC2.2 catalyzed ADP-ribosylation.

Naturally occurring Foxp3^+^ Tregs constitutively express CD25 and ARTC2.2^37^. DEREG-transgenic mice express a diphtheria-toxin receptor-GFP fusion protein under control of the foxp3 promoter, allowing visualization of living Tregs by flow cytometry^42^. The availability of ARTC2^−/−^ DEREG mice^37^ permits a direct assessment of the effect of NAD^+^-dependent ADP-ribosylation on Tregs, e.g. by comparing the responses of Tregs from ARTC2^−/−^ and wildtype DEREG mice to extracellular NAD^+^ (Fig. 5c and Supplementary Fig. 5). Treatment of FACS-sorted Tregs with extracellular NAD^+^ inhibited IL-2-induced phosphorylation of STAT5 (Fig. 5c). Similarily, treatment of unsorted splenocytes with NAD^+^ inhibited phosphorylation of STAT5 in wildtype Tregs cells but not in ARTC2^−/−^ Tregs (Supplementary Fig. 5a–c). We have previously shown that ADP-ribosylation induces gating of the P2X7 ion channel, resulting in Calcium influx and externalization of phosphatidylserine^38^. In order to determine whether the inhibition of IL-2-induced STAT5 phosphorylation was mediated by P2X7, we analyzed the effects of extracellular NAD^+^ also on cells from P2X7^−/−^ (ARTC2^+/+^) DEREG mice (Supplementary Fig. 5e). The results show that NAD^+^-treatment inhibits STAT5 phoshporylation also in purified Tregs from P2X7^−/−^ (ARTC2^+/+^ mice), indicating that this effect is independent of P2X7 activation.

## Discussion

The results presented here identify CD25, the α chain of the interleukin-2 receptor, as a major target for ADP-ribosylation by the toxin-related ecto-enzyme ARTC2.2 (Fig. 1) and provide insight into the role of ADP-ribosylation of CD25. ADP-ribosylation assays in HEK cells co-transfected with ARTC2.2 and CD25 mutants identified R35 at the arginine doublet R35R36 as a target site for ADP-ribosylation (Fig. 2). This is reminiscent of murine P2X7 and human defensin 1, both of which contain double arginines as primary sites of ADP-ribosylation, but are additionally ADP-ribosylated on other arginine residues^38^,^39^. R35 is localized within the IL-2 binding region (Supplementary Figs. 1d, 4)^7^,^43^. Considering that ADP-ribosylation results in the attachment of a bulky residue, converting a positively charged arginine into a negatively charged ADP-ribosylarginine, it is not surprising that ADP-ribosylation at this residue interferes with binding of IL-2 (Fig. 4c). Consistently, ADP-ribosylation of CD25 inhibits IL-2-induced phosphorylation of STAT5 (Fig. 5a, c) as well as IL-2-dependent cell proliferation (Fig. 4f). Of note, ADP-ribosylation of CD25 blocks binding of mAb 7D4 (Fig. 3), an antibody that is commonly used for bead-assisted sorting of Tregs. Considering that endogenous NAD^+^ can be released during manipulation of cells^37^,^44^, inadvertent ADP-ribosylation of CD25 prior to staining with 7D4 may hamper bead-assisted sorting of Tregs. Indeed, NAD^+^-mediated ADP-ribosylation of CD25 may explain the reported difficulty to purify Tregs from CD38ko mice using bead-assisted sorting^45^ since lack of the major cell surface NAD-hydrolase in these mice causes exposure of cells to higher levels of extracellular NAD^+^^37^,^46^. We recently described a simple means to prevent inadvertent ADP-ribosylation of cell surface proteins during preparation of cells from spleen or liver, i.e. systemic injection of an ARTC2.2-blocking nanobody ten minutes prior to sacrifice of mice^47^.

**Figure 6:**
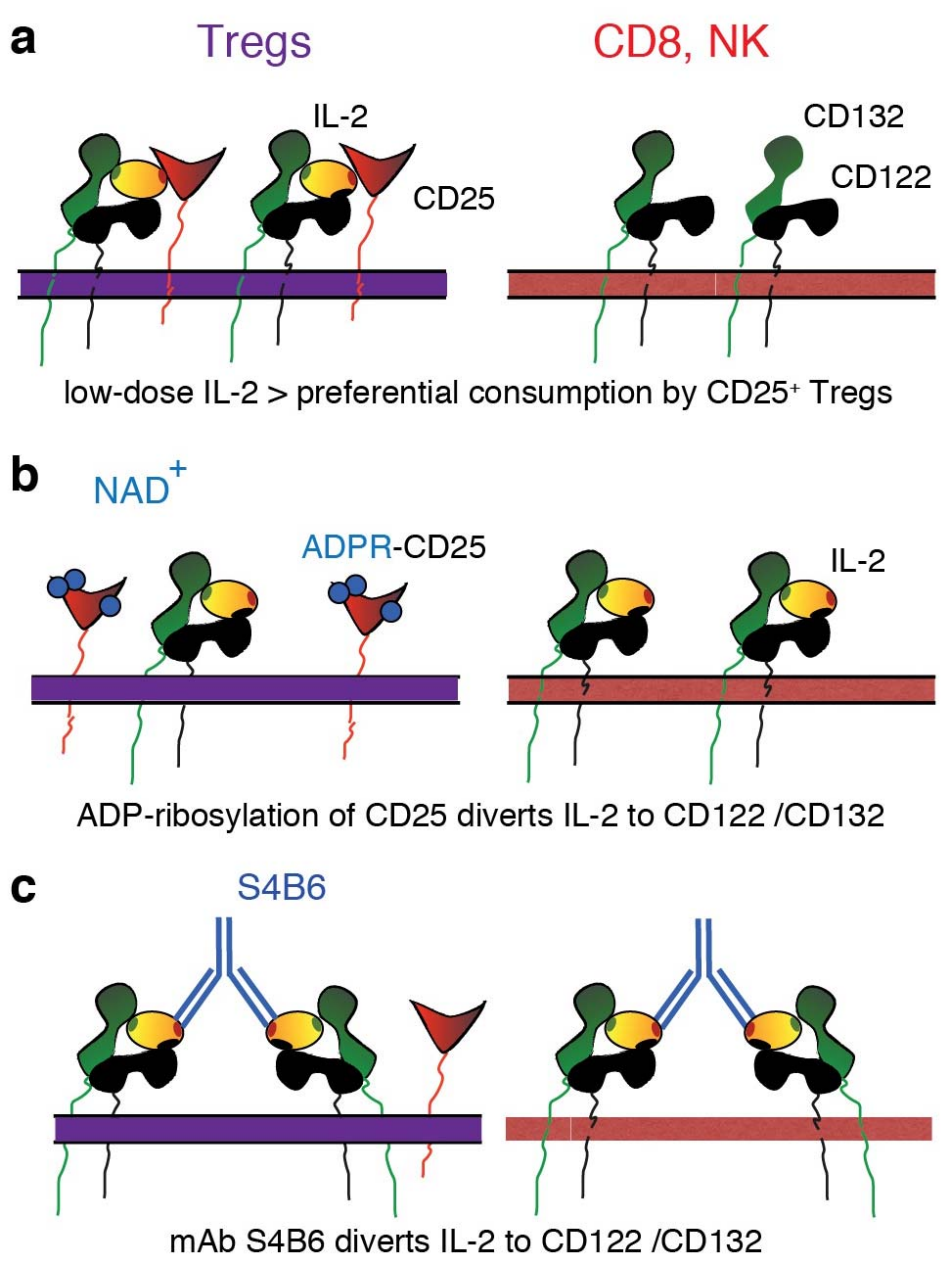
Model for the tuning of IL-2 signaling by ADP-ribosylation of CD25. (a) Tregs constitutively express high levels of CD25 while CD8^+^ T cells and NK cells express only the β and γ chains of the IL-2 receptor (CD132, CD122). In a non-inflammatory environment, Tregs consume IL-2 due to the higher affinity of CD25 compared to CD122/CD132, thereby depriving neighboring CD8 cells and NK cells of this cytokine. (b) In an inflammatory environment, i.e. following the release of NAD^+^ from damaged cells, ADP-ribosylation of CD25 (blue circles) diverts IL-2 from the high affinity receptor to the low affinity β and γ chains, allowing efficient expansion of CD8 cells and NK cells. (c) Systemic injection of IL-2 in complex with antibodies that prevent binding of IL-2 to CD25 similarly results in preferential expansion of CD8 cells and NK cells.

Figure 6 illustrates a model whereby ADP-ribosylation of CD25 provides a regulatory mechanism for tuning IL-2 signaling, i.e. from CD25-dependent high affinity signaling to CD25-independent low affinity signaling via CD122/CD132. In a non-inflammatory environment, where the concentration of extracellular NAD^+^ is low, consumption of extracellular IL-2 by Tregs would deprive other T cells and NK cells of IL-2 (Fig. 6a)^48^. Similarly, systemic injections of low doses of IL-2 may result in the preferential expansion of Tregs^17^,^19^,^20^,^21^,^49^. In an inflammatory environment, where stressed and damaged cells release large amounts of NAD^+^ into the extracellular compartment^44^,^50^, ADP-ribosylation of CD25 would divert IL-2 signaling away from Tregs to NK cells and CD8^+^ cytotoxic T cells (Fig. 6b). This mechanism of tuning IL-2 signaling may be mimicked by certain IL-2 antibodies used *in vivo* (Fig. 6c), i.e. systemic injections of mouse IL-2 in complex with mAbs S4B6 or JES6-5HA or of human IL-2 in complex with Mab-602 cause preferential expansion of NK cells and cytotoxic T cells^22^,^24^,^27^. In addition to Tregs that constitutively express CD25, CD25 is also up-regulated by activated T cells following triggering of the TCR (T cell receptor)^51^ (Supplementary Fig. 6). Since TCR triggering induces metalloprotease-mediated shedding of ARTC2.2, activated T cells are rendered resistant to ADP-ribosylation of cell surface proteins^52^. Therefore, ADP-ribosylation of CD25 may be a Treg-specific regulatory mechanism. The finding that some Tregs appear unaffected by NAD^+^ treatment (Fig. 5C, Supplementary Fig. 5) could be due to lower expression of ARTC2.2 or CD25 or due to differential localization of these antigens in the plasma membrane, e.g. inside/outside lipid rafts^8^,^35^. Future studies should address potential differences of distinct subpopulations of Tregs in their sensitivity to NAD^+^-mediated regulation of IL-2 signaling.

In summary, we have described here a previously unknown mechanism for tuning signaling by IL-2 by ADP-ribosylation of CD25. Operation of this mechanism *in vivo* likely depends on the context in which T cells are exposed to NAD^+^. Thus, at sites where NAD^+^ is released in large quantities from damaged cells, such as during a lytic viral infection, ADP-ribosylation of CD25 on Tregs would favor proliferation and function of CD8^+^ effector T cells, thereby enhancing pathogen eradication. In contrast, in healthy tissues, where little if any NAD^+^ is released, Treg function would not be inhibited by ADP-ribosylation, permitting IL-2 mediated expansion of Tregs and efficient suppression of potentially auto-reactive T cells.

## Methods

### Mice and cells

ARTC2^−/−^ mice^53^ and P2X7^−/−^ mice^54^ were backcrossed to C57BL/6 WT and DEREG mice^42^ on the C57BL/6 background for 12 generations and were maintained under specific pathogen-free conditions at the central animal facility of the UKE. All experiments were performed according to state guidelines with approval of the local institutional regulatory committee (registration numbers ORG153 and A10a). The YAC-1 lymphoma cells, kindly provided by Jürgen Löhler, Heinrich Pette Institute, Hamburg, Germany, were cultured in RPMI-1640 supplemented with 10% FCS, 2 mM glutamine, 2 mM sodium pyruvate. The IL-2-dependent CTLL-2 cell-line, kindly provided by Marc Pallardy, INSERM U 461, Châtenay-Malabry, France, was maintained in complete medium supplemented with 10 ng/ml human recombinant IL-2 (10000 U/ml, Roche) and 0.0001% 2-mercaptoethanol. CTLL-2 cells were stably transfected with an expression construct for ARTC2.2 (pME.CD8LF-ARTC2.2)^55^. Tregs were FACS-sorted as GFP^+^ cells from primary spleen cells of DEREG mice at 4°C. Alternatively, after erythrocyte lysis with Ack lysis buffer (Bio Whittaker) and depletion of B-cells with Dynabead-conjugated sheep anti-mouse IgG (Invitrogen), Tregs were positively selected by magnetic cell sorting using PE conjugated anti-CD25 (7D4) and magnetic bead-conjugated anti-PE antibodies according to the manufacturer's instructions (Miltenyi Biotec). Purity of Tregs was monitored by flow cytometry and was >90%.

### Flow cytometry

Fluorochrome-conjugated mAbs were used against mouse CD25 (PC61, 7D4) human CD25 (M-A251), CD4 (GK1.5), ARTC2.2 (Nika102), etheno-adenosine (1G4), and pSTAT5 (47). Stained cells were analyzed with a FACS Canto II (BD) and FlowJo software (TriStar).

### Site directed mutagenesis and cell transfections

The coding regions for mouse and human CD25 were PCR-amplified from YAC-1 lymphoma cells and from ConA-stimulated PBLs, respectively, and cloned into the pCMV-Sport6 vector (Invitrogen). Arginine residues were substituted with lysine using site-directed mutagenesis. A synthetic cDNA encoding CD25 in which all arginine residues in the extracellular domain were replaced by lysine, was purchased from Geneart (Invitrogen) and cloned into pCMV-Sport6. Expression constructs (2 μg per 10^6^ cells) were transfected into CTLL-2 cells by electroporation and into HEK cells with the jetPEI transfection reagent (Polyplus).

### Purification of etheno-ADP-ribosylated proteins and mass spectrometry

YAC-1 cells (10^9^ cells in 10 ml) were incubated for 15 min at RT with 10 μM etheno-NAD^+^ (Sigma) and washed twice in PBS before lysis in 1% Triton-X 100 for 20 min at 4°C. Cell lysates were clarified by high speed centrifugation (20 min 15.000 g) before passage through a 1 ml affinity column containing mAb 1G4 immobilized on Amino-Link Matrix (Pierce). The column was extensively washed with 1% Triton X-100 and PBS, bound proteins were eluted with 1 mM etheno-adenosine in PBS/0.1% Triton-X-100. Eluted proteins were size fractionated by SDS-PAGE. Proteins were stained with Coomassie blue and visible bands were cut out. A parallel gel was blotted onto a PVDF membrane and subjected to Western-Blot analyses with etheno-adenosine specific mAb 1G4 and peroxidase-conjugated anti-mouse IgG. A prominent 50 kD band was excised from the gel. Following reduction of disulfide bonds with DTT (10 mM, 56°C, 30 min) and modification of cysteines with iodoacetamide (55 mM, RT, 20 min), proteins were subjected to in-gel digestion with trypsin (5 ng/μl in 50 mM NH_4_HCO_3_, 37°C, 16 h). Peptides were extracted with 50% acetonitrile/5% formic acid, dried in a vacuum concentrater, redisolved in 5% methanol/5% formic acid desalted on a C18 μZipTip (Millipore), eluted with 1 μl 60% methanol/5% formic acid, and analyzed by nano-electrospray mass spectrometry in a QTOF II instrument (Micromass). MS/MS spectra obtained by collision induced fragmentation after manual precurser selection were evaluated both manually and by the Mascot MS/MS search algorithm version 2.2 (Matrix Sciences, London, UK).

### Radio-ADP-ribosylation assays

HEK cells were transiently co-transfected with ARTC2.2 (1 μg/10^6^ cells) and CD25 (2 μg/10^6^ cells) in solution and plated in parallel aliquots onto 6-well plates pre-coated with poly-L-lysine. 48 h post transfection, one aliquot was used to monitor transfection efficiency by FACS analyses using mAbs directed against ARTC2.2 and CD25. The adherent cells of the other aliquot were gently washed with pre-warmed serum-free X vivo medium (Bio Whittaker) and then incubated in serum free X vivo medium containing ^32^P-NAD^+^ (2,5 μCi, 0,4 μM) for 20 min at 37°C. Cells were gently washed with pre-warmed X vivo medium and then lysed in PBS, 1% Triton-X100, 1 mM AEBSF (Sigma) for 20 min at 4°C. Insoluble material was pelleted by high-speed centrifugation (15 min 13.000 g) and solubilized membrane proteins were size fractionated on precast SDS-PAGE gels (Invitrogen) (1 × 10^5^ cell equivalents/lane). Proteins were detected by Coomassie staining of the gels. For detection of radiolabeled proteins, gels were exposed to an X-ray film at −80°C for 6h–6d.

### Immunoprecipitation of CD25

The CD25-specific mAb PC61 was conjugated to amino-link beads according to the manufacturer's instructions (Pierce). After incubation with cell lysates for 60 min at 4°C, beads were washed with PBS, 1% Triton-X 100. Samples were then incubated at 70°C for 15 min and the matrix was pelleted by centrifugation. Proteins eluted from the matrix were analyzed by SDS-PAGE autoradiography as described above.

### Cell proliferation assay

In order to deplete CTLL-2 cells from IL-2, cells were washed twice and incubated for 6 h in culture medium without IL-2. Cells were seeded in a 24-well plate (3 × 10^4^ cells per well) and cultivated in medium containing serial dilutions of IL-2. A small aliquot of medium containing or lacking NAD^+^ (final concentration 100 μM) was added every 12 hours. Cell numbers were determined after four days by flow cytometry using Trucount™ beads (BD).

### STAT5 phosphorylation assay

Primary splenocytes and purified Tregs (1 × 10^5^ cells/100 μl) were incubated in the absence or presence of 30 μM NAD^+^ for 15 min at 4°C before addition of 2.5 U/ml mouse IL-2 (eBioscience) and further incubation for 5 min at 37°C. The cells were then fixed in 2% PFA for 10 min at 37°C and 90% methanol at −20°C before staining with APC-conjugated anti-phospho-STAT5 according to the manufacturer's instructions (BD). Splenocytes were counterstained with anti-CD4 before incubation with IL-2. In some experiments, cells were preincubated for 15 min in the presence of the ARTC2.2-blocking nanobody s+16a (50 ng/ml)^41^.

### Interleukin-2 binding assay

1 × 10^5^ cells were incubated with 5 ng biotinylated Interleukin-2 (R&D Systems) or 10 ng biotinylated soybean-trypsin inhibitor in 100 μl PBS, 0.1% BSA for 60 min at 4°C before addition of 100 ng APC-conjugated streptavidin (BD). Parallel aliquots of cells were pre-incubated with or without unlabeled mIL-2 (500 ng, eBioscience) or 50 μM NAD^+^.

## Author Contributions

S.T. and A.H. contributed equally. S.T., A.H., M.M., C.M.M., B.R., S.M., P.B., F.B. and M.N. performed experiments and analyzed results. S.T., B.R., S.M., P.B., F.H., O.B., S.A., M.S. and F.K.-N. designed experiments and analyzed the data. S.T. and F.K.-N. wrote the paper.

## Supplementary Material

Supplementary Information Supplementary Information

## References

[b1] Sakaguchi S. Naturally arising Foxp3-expressing CD25+CD4+ regulatory T cells in immunological tolerance to self and non-self. Nat Immunol 6, 345–352 (2005). 15785760 10.1038/ni1178

[b2] Malek T. R. & Castro I. Interleukin-2 receptor signaling: at the interface between tolerance and immunity. Immunity 33, 153–165 (2010). 20732639 10.1016/j.immuni.2010.08.004 PMC2946796

[b3] Bluestone J. A. The yin and yang of interleukin-2-mediated immunotherapy. N Engl J Med 365, 2129–2131 (2011). 22129258 10.1056/NEJMe1110900

[b4] Boyman O. & Sprent J. The role of interleukin-2 during homeostasis and activation of the immune system. Nat Rev Immunol 12, 180–190 (2012). 22343569 10.1038/nri3156

[b5] Matko J. *et al.* GPI-microdomains (membrane rafts) and signaling of the multi-chain interleukin-2 receptor in human lymphoma/leukemia T cell lines. Eur J Biochem 269, 1199–1208 (2002). 11856346 10.1046/j.0014-2956.2002.02759.x

[b6] Wang X., Rickert M. & Garcia K. C. Structure of the quaternary complex of interleukin-2 with its alpha, beta, and gammac receptors. Science 310, 1159–1163 (2005). 16293754 10.1126/science.1117893

[b7] Stauber D. J., Debler E. W., Horton P. A., Smith K. A. & Wilson I. A. Crystal structure of the IL-2 signaling complex: paradigm for a heterotrimeric cytokine receptor. Proc Natl Acad Sci U S A 103, 2788–2793 (2006). 16477002 10.1073/pnas.0511161103 PMC1413841

[b8] Marmor M. D. & Julius M. Role for lipid rafts in regulating interleukin-2 receptor signaling. Blood 98, 1489–1497 (2001). 11520799 10.1182/blood.v98.5.1489

[b9] Kovanen P. E. & Leonard W. J. Cytokines and immunodeficiency diseases: critical roles of the gamma(c)-dependent cytokines interleukins 2, 4, 7, 9, 15, and 21, and their signaling pathways. Immunol Rev 202, 67–83 (2004). 15546386 10.1111/j.0105-2896.2004.00203.x

[b10] Nelson B. H., Lord J. D. & Greenberg P. D. Cytoplasmic domains of the interleukin-2 receptor beta and gamma chains mediate the signal for T-cell proliferation. Nature 369, 333–336 (1994). 7514277 10.1038/369333a0

[b11] Bensinger S. J. *et al.* Distinct IL-2 receptor signaling pattern in CD4+CD25+ regulatory T cells. J Immunol 172, 5287–5296 (2004). 15100267 10.4049/jimmunol.172.9.5287 PMC2842445

[b12] Burchill M. A., Yang J., Vogtenhuber C., Blazar B. R. & Farrar M. A. IL-2 receptor beta-dependent STAT5 activation is required for the development of Foxp3+ regulatory T cells. J Immunol 178, 280–290 (2007). 17182565 10.4049/jimmunol.178.1.280

[b13] Nouze C., Pasquet L. & van Meerwijk J. P. In vitro expansion of alloantigen-specific regulatory T cells and their use in prevention of allograft rejection. Methods Mol Biol 707, 187–196 (2011). 21287336 10.1007/978-1-61737-979-6_12

[b14] Fontenot J. D., Rasmussen J. P., Gavin M. A. & Rudensky A. Y. A function for interleukin 2 in Foxp3-expressing regulatory T cells. Nat Immunol 6, 1142–1151 (2005). 16227984 10.1038/ni1263

[b15] Setoguchi R., Hori S., Takahashi T. & Sakaguchi S. Homeostatic maintenance of natural Foxp3(+) CD25(+) CD4(+) regulatory T cells by interleukin (IL)-2 and induction of autoimmune disease by IL-2 neutralization. J Exp Med 201, 723–735 (2005). 15753206 10.1084/jem.20041982 PMC2212841

[b16] Busse D. *et al.* Competing feedback loops shape IL-2 signaling between helper and regulatory T lymphocytes in cellular microenvironments. Proc Natl Acad Sci U S A 107, 3058–3063 (2010). 20133667 10.1073/pnas.0812851107 PMC2840293

[b17] Grinberg-Bleyer Y. *et al.* IL-2 reverses established type 1 diabetes in NOD mice by a local effect on pancreatic regulatory T cells. J Exp Med 207, 1871–1878 (2011). 20679400 10.1084/jem.20100209 PMC2931175

[b18] Tang Q. *et al.* Central role of defective interleukin-2 production in the triggering of islet autoimmune destruction. Immunity 28, 687–697 (2008). 18468463 10.1016/j.immuni.2008.03.016 PMC2394854

[b19] Koreth J. *et al.* Interleukin-2 and regulatory T cells in graft-versus-host disease. N Engl J Med 365, 2055–2066 (2011). 22129252 10.1056/NEJMoa1108188 PMC3727432

[b20] Saadoun D. *et al.* Regulatory T-cell responses to low-dose interleukin-2 in HCV-induced vasculitis. N Engl J Med 365, 2067–2077 (2011). 22129253 10.1056/NEJMoa1105143

[b21] Hartemann A. *et al.* Low-dose interleukin 2 in patients with type 1 diabetes: a phase 1/2 randomised, double-blind, placebo-controlled trial. Lancet Diabetes Endocrinol 1, 295–305 (2013). 24622415 10.1016/S2213-8587(13)70113-X

[b22] Kim M. G. *et al.* IL-2/anti-IL-2 complex attenuates renal ischemia-reperfusion injury through expansion of regulatory T cells. J Am Soc Nephrol 24, 1529–1536 (2013). 23833258 10.1681/ASN.2012080784 PMC3785269

[b23] Letourneau S. *et al.* IL-2/anti-IL-2 antibody complexes show strong biological activity by avoiding interaction with IL-2 receptor alpha subunit CD25. Proc Natl Acad Sci U S A 107, 2171–2176 (2010). 20133862 10.1073/pnas.0909384107 PMC2836659

[b24] Boyman O., Kovar M., Rubinstein M. P., Surh C. D. & Sprent J. Selective stimulation of T cell subsets with antibody-cytokine immune complexes. Science 311, 1924–1927 (2006). 16484453 10.1126/science.1122927

[b25] Kamimura D. & Bevan M. J. Naive CD8+ T cells differentiate into protective memory-like cells after IL-2 anti IL-2 complex treatment in vivo. J Exp Med 204, 1803–1812 (2007). 17664293 10.1084/jem.20070543 PMC2118678

[b26] Webster K. E. *et al.* In vivo expansion of T reg cells with IL-2-mAb complexes: induction of resistance to EAE and long-term acceptance of islet allografts without immunosuppression. J Exp Med 206, 751–760 (2009). 19332874 10.1084/jem.20082824 PMC2715127

[b27] Krieg C., Letourneau S., Pantaleo G. & Boyman O. Improved IL-2 immunotherapy by selective stimulation of IL-2 receptors on lymphocytes and endothelial cells. Proc Natl Acad Sci U S A 107, 11906–11911 (2010). 20547866 10.1073/pnas.1002569107 PMC2900642

[b28] Boyman O. *et al.* Selectively expanding subsets of T cells in mice by injection of interleukin-2/antibody complexes: implications for transplantation tolerance. Transplant Proc 44, 1032–1034 (2012). 22564618 10.1016/j.transproceed.2012.01.093

[b29] Hottiger M. O., Hassa P. O., Luscher B., Schuler H. & Koch-Nolte F. Toward a unified nomenclature for mammalian ADP-ribosyltransferases. Trends Biochem Sci 35, 208–219 (2010). 20106667 10.1016/j.tibs.2009.12.003

[b30] Hottiger M. O. *et al.* Progress in the function and regulation of ADP-Ribosylation. Sci Signal 4, mr5 (2011). 21610250 10.1126/scisignal.2001645

[b31] Vitiello L., Gorini S., Rosano G. & la Sala A. Immunoregulation through extracellular nucleotides. Blood 120, 511–518 (2012). 22661701 10.1182/blood-2012-01-406496

[b32] Haag F. *et al.* Extracellular NAD and ATP: Partners in immune cell modulation. Purinergic Signal 3, 71–81 (2007). 18404420 10.1007/s11302-006-9038-7 PMC2096762

[b33] Koch-Nolte F., Fischer S., Haag F. & Ziegler M. Compartmentation of NAD+-dependent signalling. FEBS Lett 585, 1651–1656 (2011). 21443875 10.1016/j.febslet.2011.03.045

[b34] Deaglio S. & Robson S. C. Ectonucleotidases as regulators of purinergic signaling in thrombosis, inflammation, and immunity. Adv Pharmacol 61, 301–332 (2011). 21586363 10.1016/B978-0-12-385526-8.00010-2 PMC5879773

[b35] Bannas P. *et al.* Activity and specificity of toxin-related mouse T cell ecto-ADP-ribosyltransferase ART2.2 depends on its association with lipid rafts. Blood 105, 3663–3670 (2005). 15657180 10.1182/blood-2004-08-3325

[b36] Aswad F., Kawamura H. & Dennert G. High sensitivity of CD4+CD25+ regulatory T cells to extracellular metabolites nicotinamide adenine dinucleotide and ATP: a role for P2X7 receptors. J Immunol 175, 3075–3083 (2005). 16116196 10.4049/jimmunol.175.5.3075

[b37] Hubert S. *et al.* Extracellular NAD+ shapes the Foxp3+ regulatory T cell compartment through the ART2-P2X7 pathway. J Exp Med 207, 2561–2568 (2010). 20975043 10.1084/jem.20091154 PMC2989765

[b38] Adriouch S. *et al.* ADP-ribosylation at R125 gates the P2X7 ion channel by presenting a covalent ligand to its nucleotide binding site. Faseb J 22, 861–869 (2008). 17928361 10.1096/fj.07-9294com

[b39] Paone G. *et al.* ADP-ribosyltransferase-specific modification of human neutrophil peptide-1. J Biol Chem 281, 17054–17060 (2006). 16627471 10.1074/jbc.M603042200

[b40] Laing S., Unger M., Koch-Nolte F. & Haag F. ADP-ribosylation of arginine. Amino Acids 41, 257–269 (2011). 20652610 10.1007/s00726-010-0676-2 PMC3102197

[b41] Koch-Nolte F. *et al.* Single domain antibodies from llama effectively and specifically block T cell ecto-ADP-ribosyltransferase ART2.2 in vivo. Faseb J 21, 3490–3498 (2007). 17575259 10.1096/fj.07-8661com

[b42] Lahl K. *et al.* Selective depletion of Foxp3+ regulatory T cells induces a scurfy-like disease. J Exp Med 204, 57–63 (2007). 17200412 10.1084/jem.20061852 PMC2118432

[b43] Robb R. J., Rusk C. M. & Neeper M. P. Structure-function relationships for the interleukin 2 receptor: location of ligand and antibody binding sites on the Tac receptor chain by mutational analysis. Proc Natl Acad Sci U S A 85, 5654–5658 (1988). 3135551 10.1073/pnas.85.15.5654 PMC281818

[b44] Scheuplein F. *et al.* NAD+ and ATP released from injured cells induce P2X7-dependent shedding of CD62L and externalization of phosphatidylserine by murine T cells. J Immunol 182, 2898–2908 (2009). 19234185 10.4049/jimmunol.0801711

[b45] Patton D. T., Wilson M. D., Rowan W. C., Soond D. R. & Okkenhaug K. The PI3K p110delta regulates expression of CD38 on regulatory T cells. PLoS One 6, e17359 (2011). 21390257 10.1371/journal.pone.0017359 PMC3046981

[b46] Krebs C. *et al.* CD38 controls ADP-ribosyltransferase-2-catalyzed ADP-ribosylation of T cell surface proteins. J Immunol 174, 3298–3305 (2005). 15749861 10.4049/jimmunol.174.6.3298

[b47] Rissiek B., Danquah W., Haag F. & Koch-Nolte F. Technical Advance: a new cell preparation strategy that greatly improves the yield of vital and functional Tregs and NKT cells. J Leukoc Biol 95, 543–549 (2014). 24212099 10.1189/jlb.0713407

[b48] Pandiyan P., Zheng L., Ishihara S., Reed J. & Lenardo M. J. CD4+CD25+Foxp3+ regulatory T cells induce cytokine deprivation-mediated apoptosis of effector CD4+ T cells. Nat Immunol 8, 1353–1362 (2007). 17982458 10.1038/ni1536

[b49] Churlaud G. *et al.* Sustained stimulation and expansion of Tregs by IL2 control autoimmunity without impairing immune responses to infection, vaccination and cancer. Clin Immunol 151, 114–126 (2014). 24576619 10.1016/j.clim.2014.02.003

[b50] Adriouch S. *et al.* NAD+ released during inflammation participates in T cell homeostasis by inducing ART2-mediated death of naive T cells in vivo. J Immunol 179, 186–194 (2007). 17579037 10.4049/jimmunol.179.1.186

[b51] Uchiyama T., Broder S. & Waldmann T. A. A monoclonal antibody (anti-Tac) reactive with activated and functionally mature human T cells. I. Production of anti-Tac monoclonal antibody and distribution of Tac (+) cells. J Immunol 126, 1393–1397 (1981). 6970774

[b52] Kahl S. *et al.* Metalloprotease-mediated shedding of enzymatically active mouse ecto-ADP-ribosyltransferase ART2.2 upon T cell activation. J Immunol 165, 4463–4469 (2000). 11035085 10.4049/jimmunol.165.8.4463

[b53] Ohlrogge W. *et al.* Generation and characterization of ecto-ADP-ribosyltransferase ART2.1/ART2.2-deficient mice. Mol Cell Biol 22, 7535–7542 (2002). 12370300 10.1128/MCB.22.21.7535-7542.2002 PMC135670

[b54] Labasi J. M. *et al.* Absence of the P2X7 receptor alters leukocyte function and attenuates an inflammatory response. J Immunol 168, 6436–6445 (2002). 12055263 10.4049/jimmunol.168.12.6436

[b55] Koch-Nolte F. *et al.* A new monoclonal antibody detects a developmentally regulated mouse ecto ADP-ribosyltransferase on T cells: subset distribution, inbred strain variation, and modulation upon T cell activation. J Immunol 163, 6014–6022 (1999). 10570289

