# Supplementary material for: Tuning IL-2 signaling by ADP-ribosylation of CD25
Source: Sci Rep. 2015 Mar 10;5:8959. doi: 10.1038/srep08959 (PMC4354014; doi:10.1038/srep08959)

Tuning IL-2 signaling by ADP-ribosylation of CD25

Sophie Teege[1*], Alexander Hann[1*], Maria Miksiewicz[1], Cary MacMillan[1], Björn Rissiek[1], Friedrich Buck[2], Stephan Menzel[1], Marion Nissen[1], Peter Bannas[1,2], Friedrich Haag[1], Olivier Boyer[4], Michel Seman[4], Sahil Adriouch[4], Friedrich Koch-Nolte[1]

Supplementary information

Supplementary Figure 1 | Schematic diagrams of potential target arginines in the extracellular domain of CD25 and of ADP-ribosylation assays. (**a**) 3D-cartoon model of IL-2 (yellow) in complex with the heterotrimeric IL-2 receptor complex comprised of CD25 (red), CD122 (black), and CD132 (green). (**b**) 3D-surface model of CD25 with the IL-2 binding site highlighted in yellow. The eight arginines visible in the 3D-structure are highlighted in blue and are labeled with their respective residue number; the arginine doublet R35R36 in the IL-2 binding site is highlighted in cyan. 3D-models were generated with Pymol using the coordinates of pdb file 2erj [7]. (**c, d**) Following incubation of living cells with ^{32}P-NAD^{+} (**c**) or etheno-NAD^{+} (**d**) ARTC2.2 catalyzes the transfer of ^{32}P-ADP-ribose or of etheno-ADP-ribose onto arginine residues of cell surface proteins while nicotinamide is released. In this study, ^{32}P-ADP-ribosylation of CD25 was monitored by SDS-PAGE autoradiography (the radiolabel is indicated by the blue asterisk in panel **c**). Etheno-ADP-ribosylated CD25 was affinity purified using etheno-adenosine specific mAb 1G4 conjugated to agarose beads (symbolized by the blue circle in panel **d**). The structure of ARTC2.2 resembles a pacman (pbd code 1og3); the two sushi domains of CD25 form a heart-like structure (pdb file 2erj).

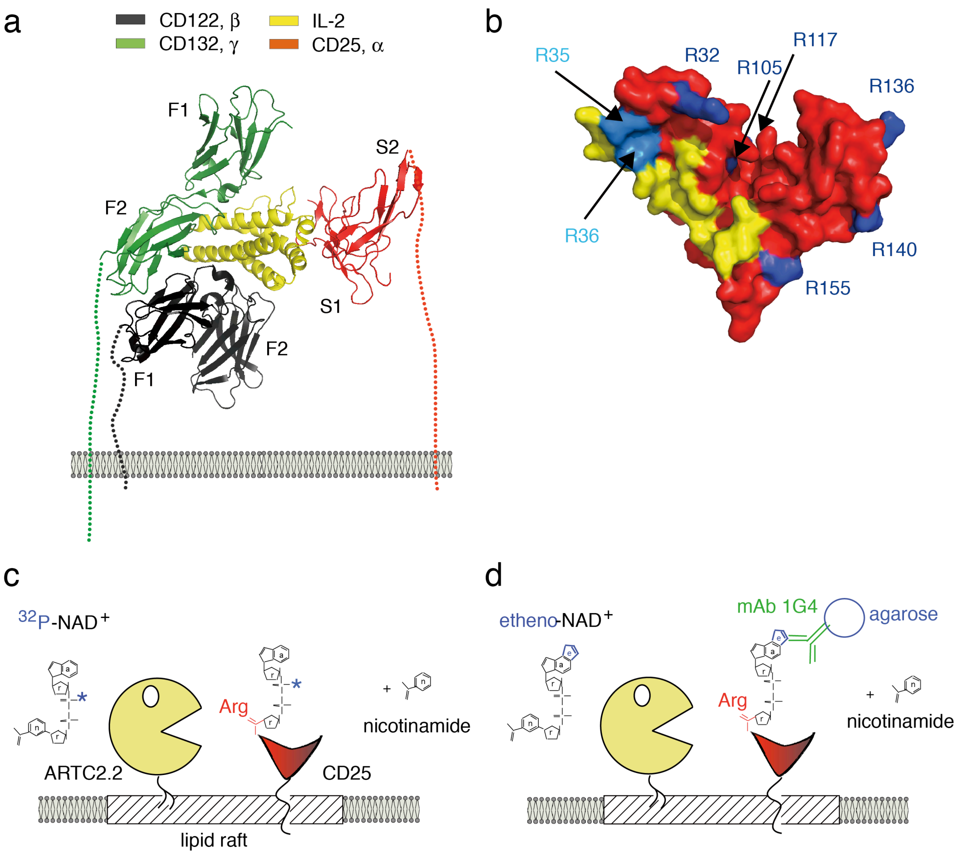

Supplementary Figure 2 | Identification of R32, R35, R67, and R140 as ADP-ribosylation sites on human CD25. HEK cells were co-transfected with expression vectors for ARTC2.2 and either wildtype CD25 (WT) or the indicated variants of CD25. 48 hours post transfection, cells were stained with PE-conjugated anti-CD25 antibody (M-A251) and APC-conjugated anti-ARTC2.2 (s+16-Fc) and cell surface expression levels were monitored by flow cytometry (**a, b**). Parallel aliquots of cells were incubated with ^{32}P-NAD^{+} and lysed in 1/% Triton-X100. Lysates were cleared by centrifugation and soluble proteins were size fractionated by SDS-PAGE (**c**). Total protein was visualized by Coomassie staining, radiolabeled proteins were detected by autoradiography. Data is from the same experiments as in Fig. 2.

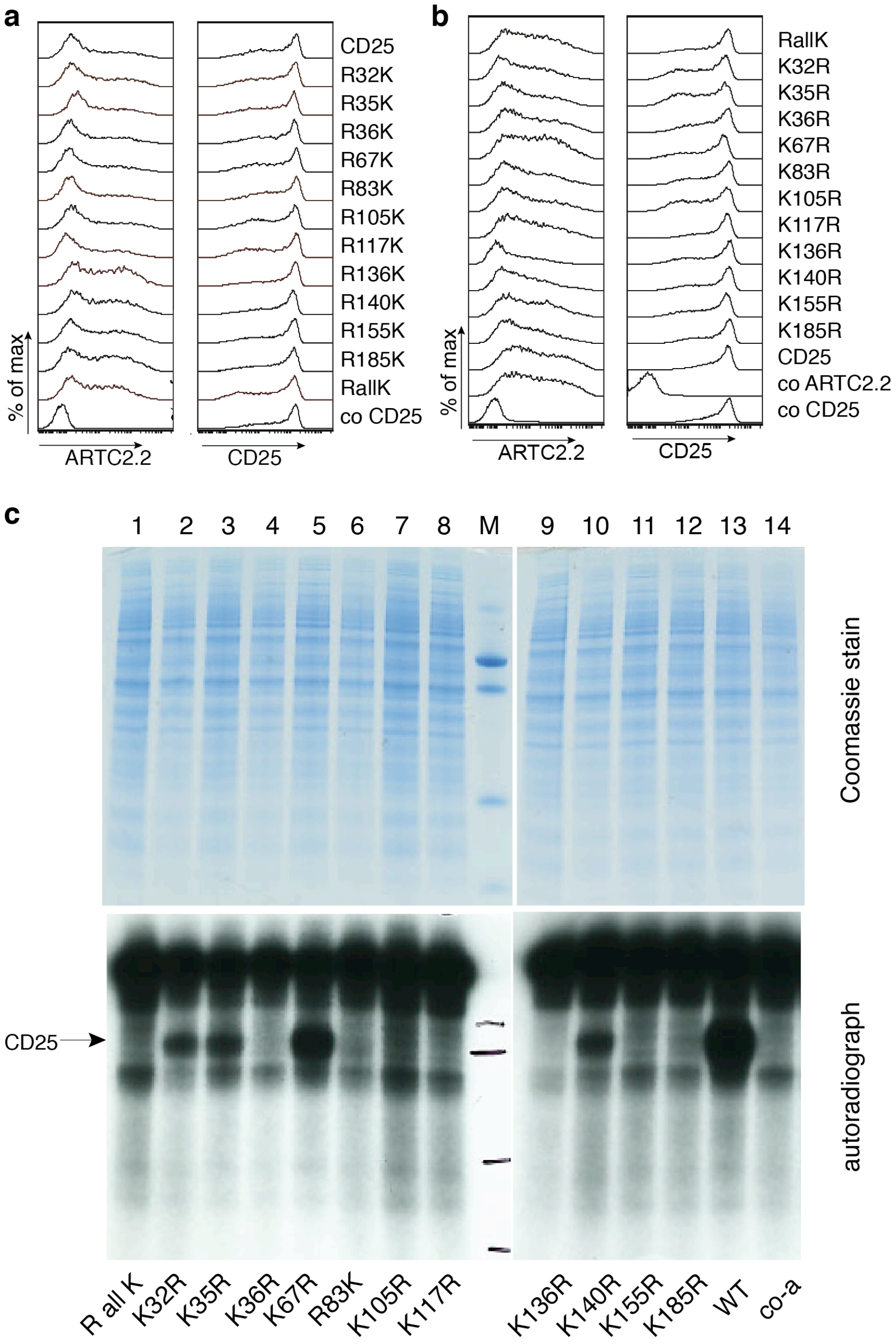

Supplementary Figure 3 | Mouse CD25 is ADP-ribosylated at more than one residue. (**a**) Schematic diagram of the 11 arginine residues in the extracellular domains of mouse CD25. Numbers correspond to the position within the amino acid sequence of the native protein, i.e. after removal of the signal peptide. Five arginines not conserved in human CD25 are underlined. Note that numbering of the conserved arginines differ in mouse and human CD25 in the second Sushi domain due to a 4 amino acid deletion in mouse *vs.* human CD25 (e.g. Fig. 2, and Supplementary Fig. 4). Dashed lines indicate residues not visible in the 3D-structure of the human IL-2 receptor complex (2erj). TMD: transmembrane domain. (**b, c**) HEK cells were co-transfected with expression vectors for ARTC2.2 and either a non-ADP-ribosylated control antigen (co), wild type CD25 (WT), or R>K mutants of mouse CD25. 48 hours post transfection, cells were stained with PE-conjugated anti-CD25 antibody (PC61) and APC-conjugated anti-ARTC2.2 (s+16-Fc) and cell surface expression levels were monitored by flow cytometry. Parallel aliquots of cells were incubated with ^{32}P-NAD^{+}. Radiolabeled proteins were detected by SDS-PAGE autoradiography. All single mutants show incorporation of radiolabel, indicating that more than one arginine of CD25 is ADP-ribosylated by ARTC2.2. Results are representative of three independent experiments.

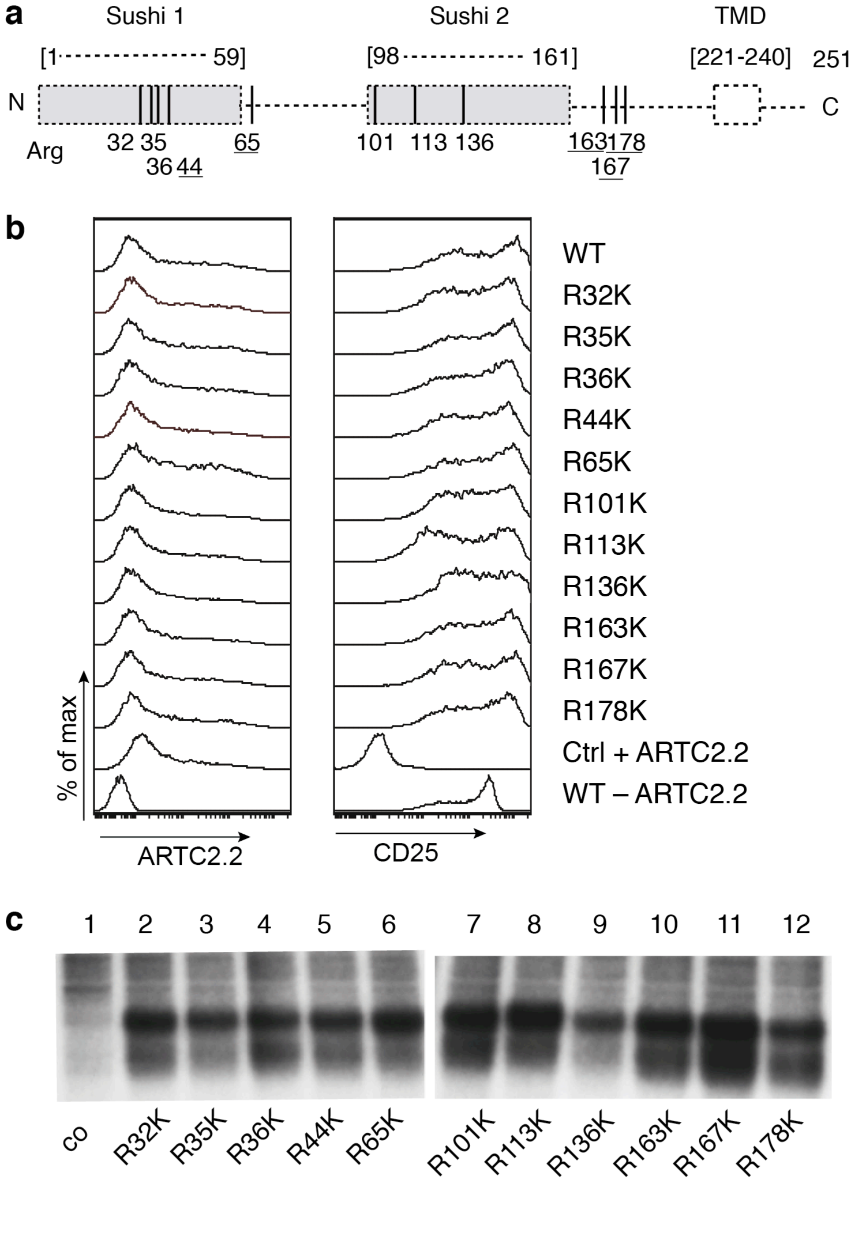

Supplementary Figure 4 | Structural context of ADP-ribosylation target arginines R32, R35, R67, and R140. (**a**) The amino acid sequences of mouse and human CD25 were aligned by T-coffee. Secondary structure units observed in the crystal structure of the IL-2 receptor complex (2erj) are indicated on top (E = beta-strands, dashes = residues not resolved in the crystal structure, numbers highlighted in yellow = disulfide bonds). The N-terminal signal peptide and the transmembrane domain are depicted in red type. Conserved arginine residues are depicted in red, non-conserved arginine residues in blue. (**b**) 3D-model of CD25. The IL-2 binding site is in yellow. Arginine residues identified as targets for ADP-ribosylation are marked in magenta (R35 within the IL-2 binding site) and in blue (R32, R140 outside the IL-2 binding site); R67 is located in a flexible region connecting the two Sushi domains and was not resolved in the crystal structure. Models were made with Pymol using the coordinates of pdb file 2erj.

a

```
       -21                  1                             32 3536
2ERJ   ---------------------  1         EEEE        EEE2 3   EEE
hCD25  MDSYLLMWGLLTFIMVPGCQAELCDDDPPEIPHATFKAMAYKEGTMLNCECKRGFRRIKS
mCD25  MEPRLLMLGFLSLTIVPSCRAELCLYDPPEVPNATFKALSYKNGTILNCECKRGFRRLKE
       -21                  1                             32 3536

       40                         67              83
2ERJ       EE4E       EE   2 3EE -----------------------------------
hCD25  GSLYMLCTGNSSHSSWDNQCQCTSSATRNTTKQVTPQPEEQKERKTT-EMQSPMQPVDQA
mCD25  -LVYMRCLGNS----WSSNCQCTSNSHDKSRKQVTAQLEHQKEQQTTTDMQKPTQSMHQE
       40   44                        65

       99   105         117                  136 140            155
2ERJ   -    4              EE      EEEEE5    EEEE    EEE1EE     EEE
hCD25  SLPGHCREPPPWENEATERIYHFVVGQMVYYQCVQGYRALHRGPAESVCKMTHGKTRWTQ
mCD25  NLTGHCREPPPWKHEDSKRIYHFVEGQSVHYECIPGYKALQRGPAISICKMKCGKTGWTQ
       95   101         113                     136

       159                      185
2ERJ     EE5E -----------------------------------------------------
hCD25  PQLICTGEMETSQFPGEEKPQASPEGRPESETSCLVTTTDFQIQTEMAATMETSIFTTEY
mCD25  PQLTCVDEREHHRFLASEESQGSRNSSPESETSCPITTTDFPQPTETTAMTETFVLTMEY
       155    163 167        178

       219
       ---------------------------------
hCD25  QVAVAGCVFLLISVLLLSGLTWQRRQRKSRRTI
mCD25  KVAVASCLFLLISILLLSGLTWQHRWRKSRRTI
       215
```

b

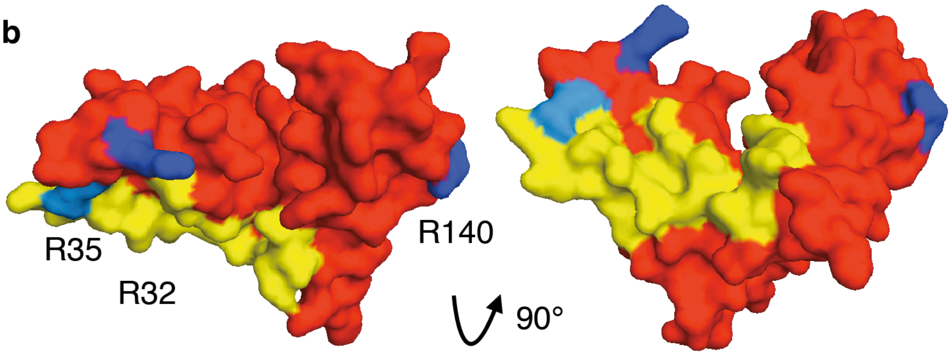

Supplementary Figure 5 | ADP-ribosylation inhibits IL-2-induced phosphorylation of STAT5 by ARTC2$^{+/+}$ but not by ARTC2$^{-/-}$ Tregs. Splenocytes were prepared from ARTC2$^{-/-}$ or WT (ARTC2$^{+/+}$) DEREG mice that express GFP under control of the Foxp3 promoter. (**a**) Cells were stained with a mAb directed against CD4 and gating was performed on CD4^{+} cells. (**b**) Cells were incubated in the absence or presence of NAD^{+} for 10 min before stimulation with IL-2 and staining for pSTAT5. Note that a small population of GFP-negative cells also responds to IL-2 by phosphorylating STAT5 in a NAD^{+}-sensitive fashion. (**c**) Gating was performed on CD4^{+}GFP^{+} cells (Tregs). (**d**) Purity of FACS-sorted Tregs from splenocytes of DEREG mice was monitored by staining with a mAb directed against CD4 and endogenous GFP fluorescence. Data is from the same experiment as in Fig. 5c. (**e**) Tregs purified by magnetic beads from ARTC2$^{-/-}$, or P2X7$^{-/-}$ (ARTC2$^{+/+}$) DEREG mice were incubated in the absence or presence of NAD^{+} before stimulation with IL-2 and staining for pSTAT5. Purity of sorted Tregs was monitored by staining with mAbs directed against CD4 and CD25.

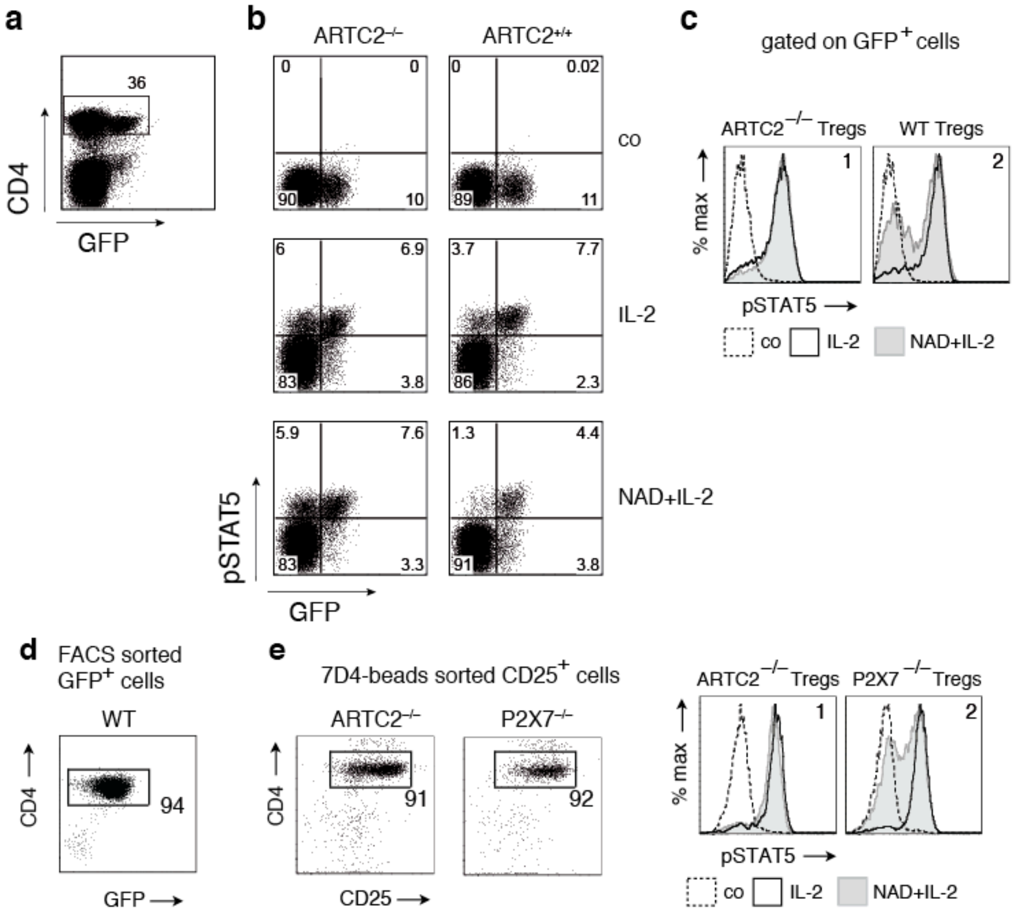

Supplementary Figure 6 | Model of CD25 ADP-ribosylation as a Treg-specific regulatory mechanism. (**a**) In a non-inflammatory environment and in the presence of low concentrations of IL-2, IL-2 is consumed mainly by Tregs which constitutively express CD25, the high affinity IL-2 receptor. IL-2-depletion prevents the proliferation of naïve effector T cells (Teff) which lack CD25. (**b**) In an inflammatory environment, i.e. following the release of NAD^+ from damaged cells, ARTC2.2-catalyzed ADP-ribosylation of CD25 (indicated by blue dots) blocks the binding and thereby the consumption of IL-2 by Tregs. Triggering of the T cell receptor on effector T cells induces shedding of ARTC2.2 and up-regulation of CD25, thereby allowing efficient expansion of effector T cells.

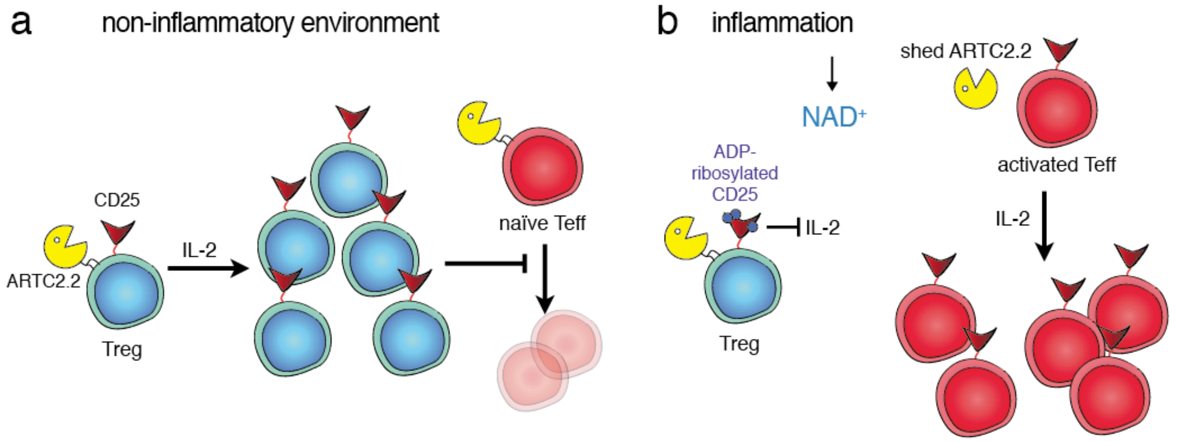

Supplement: Supplementary Information [file srep08959-s1.pdf]
